# Secondary structure in the target as a confounding factor in synthetic oligomer microarray design

**DOI:** 10.1186/1471-2164-6-31

**Published:** 2005-03-08

**Authors:** Vladyslava G Ratushna, Jennifer W Weller, Cynthia J Gibas

**Affiliations:** 1 Department of Biology, Virginia Polytechnic Institute and State University, Blacksburg, Virginia, 24061, USA; 2 School of Computational Science, Prince William Campus of George Mason University, Manassas, Virginia, 20110, USA

## Abstract

**Background:**

Secondary structure in the target is a property not usually considered in software applications for design of optimal custom oligonucleotide probes. It is frequently assumed that eliminating self-complementarity, or screening for secondary structure in the probe, is sufficient to avoid interference with hybridization by stable secondary structures in the probe binding site. Prediction and thermodynamic analysis of secondary structure formation in a genome-wide set of transcripts from *Brucella suis 1330 *demonstrates that the properties of the target molecule have the potential to strongly influence the rate and extent of hybridization between transcript and tethered oligonucleotide probe in a microarray experiment.

**Results:**

Despite the relatively high hybridization temperatures and 1M monovalent salt imposed in the modeling process to approximate hybridization conditions used in the laboratory, we find that parts of the target molecules are likely to be inaccessible to intermolecular hybridization due to the formation of stable intramolecular secondary structure. For example, at 65°C, 28 ± 7% of the average cDNA target sequence is predicted to be inaccessible to hybridization. We also analyzed the specific binding sites of a set of 70mer probes previously designed for *Brucella *using a freely available oligo design software package. 21 ± 13% of the nucleotides in each probe binding site are within a double-stranded structure in over half of the folds predicted for the cDNA target at 65°C. The intramolecular structures formed are more stable and extensive when an RNA target is modeled rather than cDNA. When random shearing of the target is modeled for fragments of 200, 100 and 50 nt, an overall destabilization of secondary structure is predicted, but shearing does not eliminate secondary structure.

**Conclusion:**

Secondary structure in the target is pervasive, and a significant fraction of the target is found in double stranded conformations even at high temperature. Stable structure in the target has the potential to interfere with hybridization and should be a factor in interpretation of microarray results, as well as an explicit criterion in array design. Inclusion of this property in an oligonucleotide design procedure would change the definition of an optimal oligonucleotide significantly.

## Background

Sequence-specific hybridization of a long single-stranded labeled DNA or RNA target molecule to shorter oligonucleotide probes is the basis of the gene expression microarray experiment. In this type of microarray experiment, gene specific *probe *molecules are either synthesized in situ or are printed to the microarray slide, and are either non-specifically cross-linked to the surface or are attached specifically using a method such as poly-Lysine linkers. *Target *molecules (most often fluorescently labeled cDNA molecules, although cRNA and aRNA are used in some protocols) hybridize transiently to the probe oligomers until they form stable double helices with their specific probes. At some point, the rate of on and off reactions reach equilibrium, and the concentration of the target in the sample solution can be calculated. Transcript abundance is assessed by the relative intensity of signal from each spot on the array. This interpretation of array data relies on the assumption that each hybridization reaction goes to completion within the timeframe of the experiment and that the behavior of all pairs of intended reaction partners in the experiment is somewhat uniform.

There are three major types of DNA microarrays, which differ in the approach used for probe design: Affymetrix type microarrays [1], which assay each transcript with a distributed set of 25-mer oligonucleotides, full length cDNA microarrays, in which long cDNA molecules of lengths up to several hundred bases are crosslinked to the slide surface to probe their complement [2], and synthetic long-oligomer probe microarrays, which usually assay each transcript only once. The latter class of microarrays encompasses a variety of commercial and custom platforms, and there has yet to emerge a consensus on an optimal probe length for particular experimental designs. Oligo lengths ranging from 35 to 70 nucleotides have been shown to perform well under different conditions [3-5], though recent studies have shown that oligomers of up to 150 nucleotides may be desirable for assessing transcript abundance [6]. In general, the use of synthetic oligomers has been shown to result in improved data quality [7,8] relative to cDNA arrays, and 70mers have been shown to detect target with a sensitivity similar to that of full length cDNA probes [9]. Short probes have been promoted because they facilitate finding unique sequence matches while forming fewer, and less stable, hairpin structures and because they display more uniform hybridization behavior overall. However, the need for sensitivity and detection of transcripts in low copy number drives the use of long-oligonucleotide arrays. In this study, we have modeled the accessibility of transcripts to hybridization with 70mer oligonucleotides.

A number of oligonucleotide design software packages have been published in recent years, each having design strengths in one of a number of criteria [10-14]. Several factors are considered by almost all microarray design software packages: in particular, the sequence specificity of the probe-target interface and the overall balance of GC content across the array. Unique regions of the target sequence are identified using sequence comparison methods; the unique regions become the search space for probe selection based on other criteria. The number of probes per sequence and location of the probe in the sequence also restrict sequence availability. A relatively uniform melting profile generally is achieved simply by selecting for probes with similar GC content and uniform or close-to-uniform length, although some design methods explicitly compute the duplex melting temperature for each candidate probe-target pair and filter unique probes to find those which match a specified range of melting temperatures. Another biophysical criterion that is sometimes applied is the elimination of probes having the ability to form stable intramolecular structures under the conditions of the experiment. This is usually done by eliminating regions of self-complementarity, although at least one design program [13] does explicitly compute the melting temperature of the most stable structure to form in the probe molecule and uses that information to filter out stable secondary structures in the probe.

**Figure 1:**
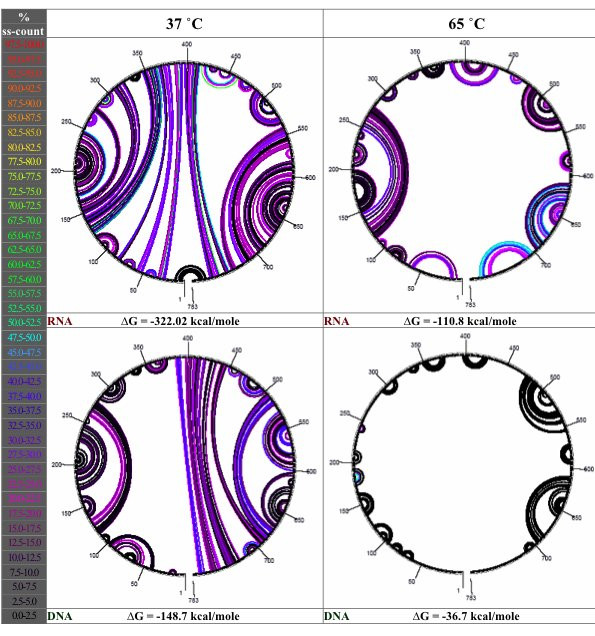
**Secondary structure in a sample transcript. **Circular diagrams of structure in a sample transcript (moeB homolog designated BR0004) from *Brucella suis*. Circular diagrams show hydrogen bonds between individual nucleotides, color-coded according to single-strandedness – the fraction of structures in which that bond is not present. Black bonds indicate 0% single-strandedness; red bonds indicate 100% single-strandedness.

Few of the available array design packages explicitly consider the possible structures of the transcript-derived molecules in the sample solution and their impact on whether the microarray will provide an effective assay, although the OligoDesign web server [14] does compute this information for use in design of locked nucleic acid probes. It has been shown that a hairpin of as little as six bases in an oligonucleotide can require a 600-fold excess of the complementary strand to displace the hairpin even partially [15]. Since the target molecules are generally longer than the probe and may be of a different chemistry, it is not sufficient to conclude that their behavior will mirror that of the complementary probe. Prediction of secondary structure in a sample transcript using a standard nucleic acid secondary structure prediction algorithm (Mfold) demonstrates that while longer-range interactions are reduced at high temperatures, stable local structures persist in the transcript even at high salt concentration and high temperature (Figure 1). Because unimolecular reactions within the target can occur on a much shorter timescale than the diffusion-mediated, bimolecular, duplex hybridization reaction, competition for binding by intramolecular structures is expected to block the specific probe annealing sites on the target sequence in some cases and result in misinterpretation of the signal obtained from the assay if these effects are not taken into account.

In order to estimate the prevalence of stable secondary structure in long target molecules, and thus the impact such structures might have on the analysis of microarray data, we have modeled secondary structure formation in mRNA transcripts of the intracellular pathogen *Brucella suis*. We have assessed the stability of structures formed in the transcript and the accessibility of the binding sites of optimal probes generated using commonly applied design criteria. Because random shearing of the full-length target molecule is used in some protocols, we have also modeled the effects of shearing to an average length on the prevalence of secondary structure in selected targets.

## Results

### Extent and stability of target secondary structure

**Figure 2:**
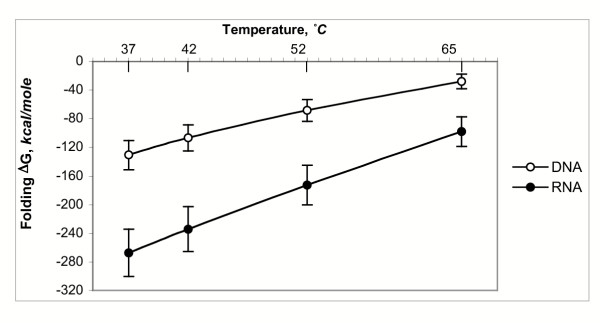
**Stability of transcript secondary structure in *Brucella suis. ***Average free energy change on global secondary structure formation for *Brucella suis *targets, modeled as DNA or RNA. ΔG values are normalized to global mean target length.

Our modeling results obtained for the genome-wide set of intact single-stranded DNA or RNA targets demonstrate that stable secondary structures are widespread in target mixtures from *Brucella suis *(Figure 2) and in randomly chosen transcripts from the genomes of *E. coli *and *L. lactis*. Figure 2 shows the ΔG of formation for the most stable predicted secondary structure of the full-length transcript, as a function of reaction temperature. The major energy components of the Mfold ΔG are hydrogen bond energy and base pair stacking energy. These can be assumed to have a roughly linear relationship with transcript length. In order to make energies from different-length transcripts comparable, energies were normalized by computing a per-residue folding ΔG for each transcript and then multiplying that value by the global mean target length, for all transcripts considered from all organisms, of 851 bp. Average ΔG of secondary structure formation decreases with increasing temperature, but even at 65°C, the average ΔG of secondary structure formation for a full-length transcript is -98.2 kcal/mol (-27.9 kcal/mol when modeled as cDNA), meaning that the transcript is quite stable in that structure and a considerable energy input will be required to displace or melt the remaining structure. The trend in ΔG of secondary structure formation from the high-GC genome of *B. suis *to the low-GC genome of *L. lactis *is a decrease in overall stability. The average normalized ΔG of secondary structure formation for transcripts selected from the GC-balanced genome (*E. coli*) is near 70% of the average for *Brucella*, while the average ΔG for transcripts from the GC-poor genome (*L. lactis*) are even lower (30% at 52°C). However, even in the most GC-poor genome, stable target secondary structure in the single-stranded target is widespread.

**Figure 3:**
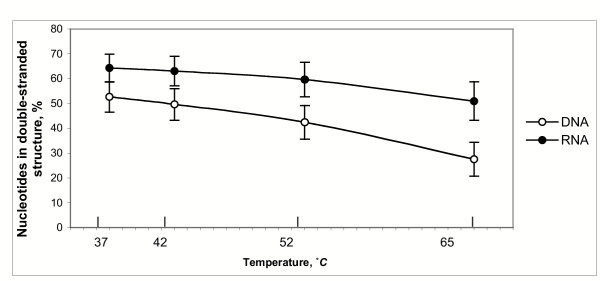
**Fractional accessibility of nucleotides in the target. **Fraction of the complete transcript classified as inaccessible due to the presence of stable structure in >50% of predicted conformations. Data shown are for 37, 42, 52 and 65°C simulations in *Brucella suis*.

Our results demonstrate that a significant fraction of nucleotide sites in the average target mixture, whether single stranded DNA or RNA, will be found in stable secondary structure under the hybridization conditions used in oligonucleotide microarray experiments, and will be relatively inaccessible for intermolecular interactions. Figure 3 shows the percentage of nucleotides that are in a double-helical state in at least 50% of the secondary structure conformations predicted by Mfold, at various reaction temperatures. The measure of accessibility used is the fraction of structures in which a nucleotide is found in a single-stranded conformation, when all optimal and suboptimal structures predicted are considered.

### Extent and stability of target secondary structure

**Figure 4:**
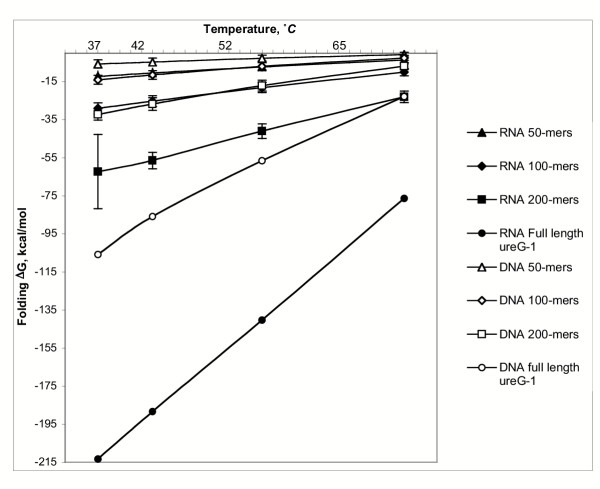
**Stability of secondary structure in sheared fragments. **Free energy change on secondary structure formation for the ureG-1 RNA transcript from *Brucella suis*. The transcript is modeled as sheared into fragments of length 200 nt, 100 nt or 50 nt; fragments are chosen starting at every 10th residue.

Figure 4 is a plot of the average ΔG of structure formation when shearing of the target molecule is simulated by dividing the target into overlapping 200, 100, and 50mer fragments. Shearing the target into smaller fragments destabilizes secondary structure, especially at very short fragment lengths. However, shearing does not eliminate occlusion of nucleotides by secondary structure, even in the shortest fragments examined. When a DNA target is modeled at 52°C, for example, the double stranded fraction decreases by only about 30% – from 41% to 29% – when the target is simulated as sheared into 50mer fragments. However, in hybridization experiments involving low copy number targets and longer oligos, creating extremely short target fragments may reduce or eliminate the signal on the chip, because the target can not be sheared specifically to present an unbroken hybridization site for the probe, and so some fragments will be created that match the probe only partially.

### Interference of secondary structure with the hybridization site

**Figure 5:**
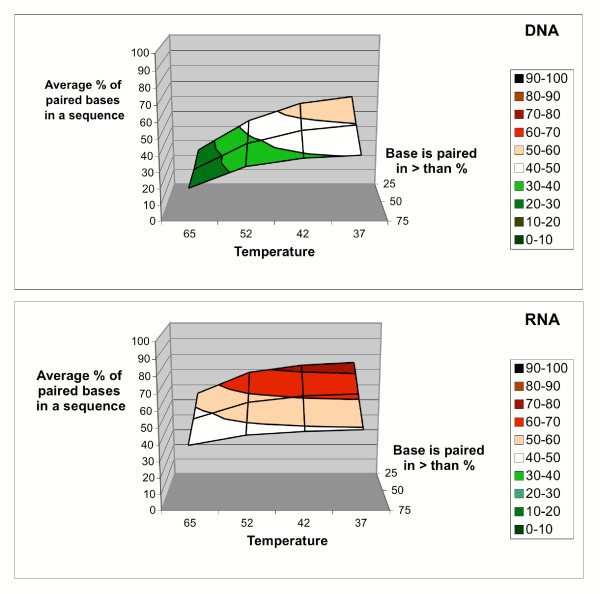
**Accessibility of the probe binding site. **Fraction of the average probe binding site in the *Brucella *genomic array that is found to be inaccessible at 37°, 42°, 52° and 65°C, for DNA or RNA target. Inaccessible sites are defined here using three different cutoffs for the fraction of structures in which the site is base-paired: 25%, 50%, and 75%.

**Figure 6:**
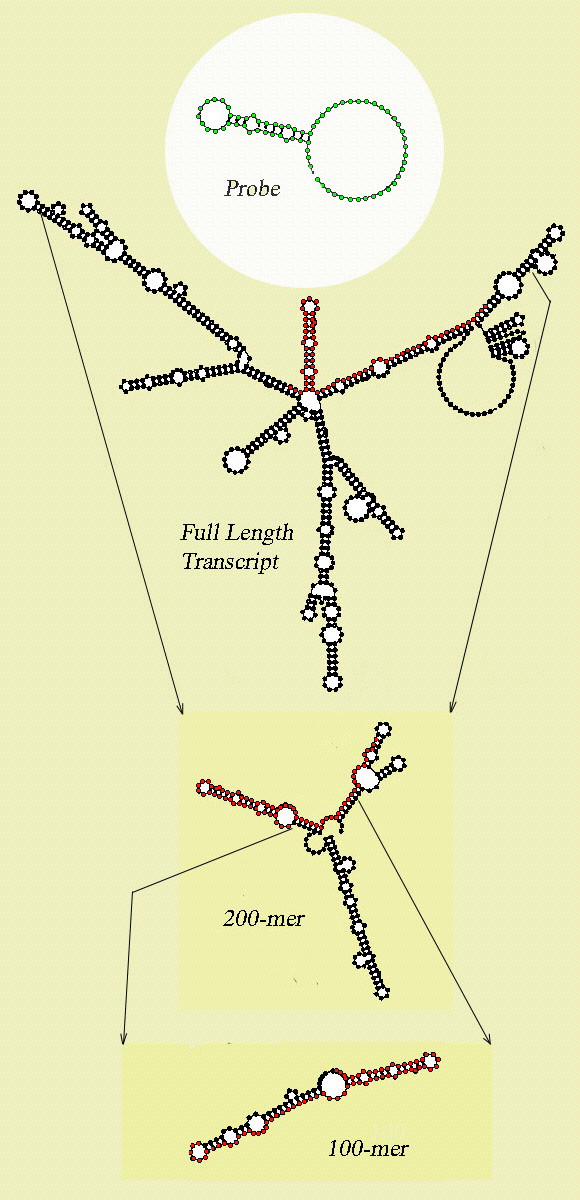
**Structure in a binding site – full length target and sheared fragments. **The position of a 70mer oligonucleotide probe (green) binding site (red dots) within a full-length optimal transcript structure, as well as examples of stable structure in 200mer and 100mer fragments which overlap the probe binding site. Corresponding ΔG values for these fragments modeled at 42° and 52°C are shown in Table 1.

Figure 5 shows the average percentage of nucleotides within a probe binding region in the target that are inaccessible, when different fractional accessibility cutoffs are used to classify the sites. Even when a relatively demanding criterion – double-strandedness in over 75% of optimal and suboptimal structures – is used to classify a nucleotide as inaccessible, an average of 21 ± 13% of nucleotides in the probe binding region are found in stable secondary structures at 65°C. Figure 6 shows a representative transcript and the challenge it presents to hybridization when modeled as full-length cDNA and fragments of various lengths.

## Discussion

Lack of bioinformatics tools that incorporate experimentally validated biophysical properties of nucleic acids as a criterion for synthetic oligomer probe design is a major challenge for do-it-yourself microarray designers. One biophysical characteristic, which we predict will reduce the binding efficiency of microarray probes to their targets, is the propensity of long single-stranded DNA or RNA molecules to form stable secondary structure. 3-D structures such as hairpins and stacked regions have the potential to pre-empt target nucleotides, thus blocking regions of the target molecules from hybridizing to their intended probes. Prediction and thermodynamic analysis of secondary structure at a range of temperatures in full length target sequences, as well as in subsequences formed by *in silico *shearing, revealed the likely presence of stable secondary structures in both full-length target and sheared target mixtures. These structures do not convert completely to random coil with either increasing hybridization temperature, more extensive shearing, or both. These secondary structures may therefore compete with the intended target for effective probe annealing in a microarray experiment, resulting in a misinterpretation of the amount of target present in the sample.

### Applying target secondary structure as a criterion in array design

Based on the results of this *in silico *experiment, secondary structure prediction in the target is being used to develop a new criterion for oligonucleotide probe design. Our results from this modeling experiment demonstrate that the implicit assumption used until now – that eliminating probe secondary structure by avoiding self-complementarity eliminates target secondary structure as well – is valid only when the target and probe are of the same length. Use of target secondary structure as an explicit criterion will allow for masking or preferentially avoiding the regions of the target sequence in which base pairs are directly involved in secondary structure formation, to eliminate these regions from the sequence for the purpose of the search for the optimal probe.

In this study we have assigned accessibility scores to sites in the target sequence based only on the fraction of predicted structures within 5% of the energy optimum, in which a residue is found in a single-stranded conformation. While this measure is not too computationally intensive to compute, and can be applied to genome-scale problems using readily available software (Mfold), it is not the most physically rigorous definition of accessibility. By equally weighting each possible structure in the ensemble of optimal and suboptimal structures that a molecule can form, it is possible that secondary structure at some positions in the molecule is overcounted; bonds which form only in rare conformations are considered equal to bonds which are present in the lowest-energy structure. The program Sfold [16-18] assigns accessibility based on an ensemble-weighted average of secondary structure. The program RNAfold[19], part of the Vienna RNA package, implements McCaskill's partition function approach[20] to arrive at pairing probabilities for each pair of bases in the sequence, from which a summary per-base accessibility can be derived. These methods are more rigorous than MFold and we expected they might produce somewhat different results, although it has also been shown that predicted binding states from MFold optimal structures perform almost as well as SFold and RNAFold predictions when applied to molecules of known 3D structure [16].

**Figure 7:**
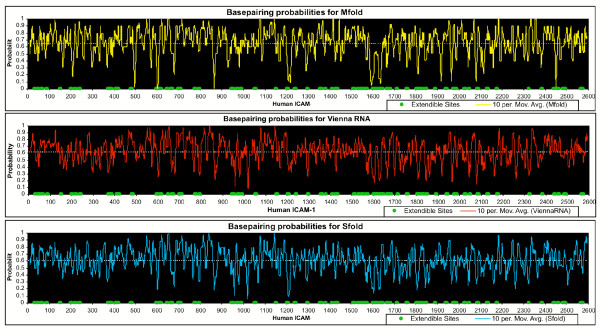
**Accessibility prediction using three common methods. **Pairing probabilities computed using RNAFold (top), MFold (middle) and SFold (bottom) for the human ICAM-1 transcript. Extendable sites detected by Allawi et al [21]

When we compared MFold-based accessibility predictions for an individual transcript to those generated by SFold and RNAFold, we found that the difference in average predicted accessibility over an entire transcript is small. We computed accessibility for the transcript of human 1CAM-1, which has been mapped experimentally to determine its accessibility [21]. The average fractional accessibility derived from MFold results is about 3–4% greater than that predicted by RNAFold or SFold. Therefore use of this fractional accessibility measure will not impose an unnecessary constraint on the design process relative to other predictive approaches. The accessibility profiles calculated for ICAM-1 using each method are shown in Fig. 7. In each section of the figure, antipeak locations (having lower pairing probability and therefore likely to be more accessible) can be compared to the extendable sites detected by Allawi et al [21], which are indicated by green dots at the bottom of the plot. In each prediction, there are a number of apparently correct predictions and obvious errors, and it is not clear which method is yielding the best results at the residue level. A systematic, competitive test of these predictions against solution accessibility data gathered on various experimental platforms is called for, although available data sets for validation are still rare. In the absence of such validation, the MFold accessibility predictions are sufficient to predict the scope of the secondary structure problem in a genome-based array design, even if some details of the prediction are not correct. An experimental approach will eventually be required to determine which approach best represents the conditions of the microarray experiment.

### Loop length and other considerations

In this study, we focused specifically on the DNA/RNA base pairs that are actively involved in hydrogen bond formation. We realize that other accessibility considerations will have to be added to the scoring scheme in practice. The structure of a long single stranded DNA or RNA molecule can contain many nucleotides that, while not part of a double-helical stem, remain inaccessible to hybridization due to their location inside small loops within the target secondary structure. A loop is a somewhat constrained structure as well, and the length at which it presents accessible sequence that favors hybridization has been shown to be on the order of 10 nucleotides and longer [22], while nucleotides found in shorter loops may be classifiable as inaccessible. However, there is a need for quantitative hybridization experiments that would elucidate how loops and loop-like structures in tethered long-oligo probe and target molecules affect the performance of assays, and we have chosen not to formulate a system for scoring the accessibility of single-stranded loop structures or weighting this criterion relative to the double-strandedness criterion until we have carried out some of these experiments.

Development of a target secondary structure criterion for oligonucleotide array design is expected to impose restrictions on the probe selection beyond the sequence similarity and melting temperature criteria that are currently used, especially in cases where short probe length restricts the annealing temperature used in the hybridization protocol to 22–37°. In the *B. suis *example, use of a low annealing temperature, e.g. 42°C which is the temperature used in some published 70-mer array experiments [9], would result in only about 30% of the average transcript being accessible for intermolecular hybridization, not counting 'free' bases found in short loops in secondary structures. There will be greater design latitude for experiments carried out at higher hybridization temperatures. Recommended hybridization temperatures for long synthetic oligomer arrays may prove to be closer to 65°C, when only 50% of a typical RNA transcript or 30% of the corresponding cDNA molecule remain inaccessible.

### To shear or not to shear

We have shown here that while shearing reduces overall ΔG of secondary structure formation for individual molecules in the target solution, shearing does not in itself eliminate formation of secondary structure in single-stranded DNA or RNA. The question of whether shearing should be used for long oligomer arrays is still an open one. While some signal may be gained by reducing the stability of secondary structure in the target molecule, random shearing by its nature creates a mixture of targets that may have substantially different affinities. For instance, in a 300 nt transcript that is targeted by a 70mer oligonucleotide, there is nearly a one in four chance that a random break in the sequence will occur within the target site for which the probe is designed. Short fragments may present a substantially different binding site, and therefore have a different binding affinity, than the full-length transcript that is considered when the probe is designed. This is illustrated in Figure 8d, where binding of a 50mer sheared fragment to a 70mer probe leaves a dangling end in the probe. A break very close to one end or the other of the target site may create a target that still binds to the probe, though with reduced affinity; a break closer to the middle of the target site may produce fragments that bind partially to the probe, competing for binding with perfect matches.

### The utility of experimentally validated biophysical criteria

In other experimental contexts where hybridization is critical to success, the impact of secondary structure in single stranded polynucleotides on results has been recognized and is now being systematically studied (18–21). Intramolecular folding of mRNAs is so extensive that only 5–10% of most transcripts is accessible to binding of complementary nucleic acids; however the modeling of long molecules has not proven to give very accurate binding predictions [23-25]. In fact, array-based screens have been utilized to empirically select oligonucleotides that bind effectively to transcripts for siRNA experiments [23,26]. Several studies have demonstrated that, at 37°C and 0 mM Mg2+ oligonucleotides of length >20 yield good binding/RNAseH digestion at low concentrations relative to shorter oligonucleotides (30 nM vs 300 nM compared) and found that microarray binding was a good predictor of siRNA activity despite the 3' tethering and 1M NaCl used in array experiments vs siRNA experiments [26]. Systematic "scanning" of mRNA sequences with libraries of short oligos [27] has also been shown to be successful in locating sites for siRNA targeting; however, such methods are likely to become extremely expensive if applied to the large number of targets in a microarray design. We have begun to develop an experimental approach to this problem, in which structure predictions like those used in this study are experimentally evaluated to determine whether the structures we can predict using existing modeling approaches will detectably affect signal in the microarray context.

## Conclusion

The results of the current study suggest a significant role for target secondary structure in hybridization to oligonucleotide arrays, which will warrant further investigation. Oligonucleotide probe binding sites in a significant fraction of transcripts are found in double-stranded conformations even in cases where self-complementarity was avoided during the probe design process. We find that at 52°C, for example, approximately 57% of probes designed for *Brucella *had binding sites in the target which were predicted to contain a stretch of unpaired bases of at least 14 nt in length; at 65°C, that fraction increased to 93%. Based on these findings we would expect that at 52°C only 57% of our probes would encounter optimal conditions for hybridization and therefore would demonstrate the expected behavior in the experiment, where intensity is expected to scale with target concentration. We predict that the remaining probes, which have shorter, or no, accessible sequences, will exhibit modified binding behavior, and we plan to conduct experiments to characterize this behavior. We have shown conclusively that avoiding self-complementarity in the probe when designing an oligonucleotide array is insufficient to eliminate secondary structure from the binding site in the target. By combining the procedure for systematic computational assessment of transcript accessibility described in this study with selective experimental validation of the impact of predicted accessibility on hybridization, we will develop a useful criterion for avoiding troublesome secondary structure when designing microarray targets.

## Methods

Prediction and thermodynamic analysis of secondary structure was performed for all protein-coding gene transcripts predicted from 3264 CDSs in the *Brucella suis *1330 genome. *Brucella suis *has a relatively high (57%) genomic GC content. *Brucella suis *was chosen for this experiment because our collaborators have previously acquired a custom synthetic oligomer microarray for this organism, developed using standard oligo array design software, and we have access to both target sequences and to a set of unique probe sequences that define the interaction sites for which expression results have been obtained by the laboratory.

In order to determine whether Brucella sequences form atypical structures we randomly picked and analyzed 50 gene coding sequences from a compositionally balanced genome (*Escherichia coli*), and 50 from the GC-poor genome of the nonpathogenic AT-rich gram-positive bacterium *Lactococcus lactis *(35% genomic GC content). The *Brucella suis *genes ranged in length from 90 to 4,803 bp, with an average transcript length of 851 bp. The *E. coli *genes ranged in length from 140 to 2,660 bp, with an average transcript length of 792 bp. The range of GC content in the genes chosen was 37% to 57% with an average value of 50%, which is reasonably representative of the *E. coli *genome. The *L. lactis *genes chosen ranged in length from 140 to 2,730 bp, with an average transcript length of 765 bp., and ranged in GC content range from 30% to 42% with an average value of 35%.

### Microarray design

70-mer probes for each *Brucella suis *target were previously designed (Stephen Boyle, personal communication) using ArrayOligoSelector (pick70) [10]. ArrayOligoSelector uses sequence uniqueness, self-complementarity, and sequence complexity as criteria but does not explicitly evaluate ΔG of secondary structure formation for the probe. 72% of the probes designed using this method were found to contain secondary structures with melting temperatures greater than 65°C, and 10% contained secondary structures with melting temperatures greater than 80°C. The Brucella probes defined the interaction sites within the target transcripts for which structural accessibility was evaluated.

### Secondary structure prediction

Probe and transcript secondary structure were predicted using the Mfold 3.1 software package [28,29]. Mfold identifies the optimal folding of a nucleic acid sequence by energy minimization and can identify suboptimal foldings within a specified energy increment of the optimum as an approach to modeling the ensemble of possible structures that a single-stranded nucleotide molecule can assume. We modeled secondary structure in the single-stranded target, modeling the target both as DNA and as RNA, at a range of temperatures which is inclusive of hybridization temperatures commonly used in microarray protocols: 37°C, 42°C, 52°C and 65°C. The modeling conditions were chosen within the allowed settings of Mfold to approximate a microarray experiment: solution conditions of 1.0 M sodium concentration and no magnesium ion were used. The free energy increment for computing suboptimal foldings, ΔΔG, was set to 5% of the computed minimum free energy. The default values of the window parameters, which control the number of structures automatically computed by Mfold 3.1, were chosen based on the sequence length. Free energy changes on formation of secondary structure were extracted from the Mfold output.

### Accessibility calculation

Accessibility in folded single-stranded DNA or RNA has recently begun to be addressed in a few experimental studies, mainly with the goal of targeting appropriate sites for RNAi. Because the structure of single-stranded nucleotide molecules is much more dynamic than that of proteins, with each molecule likely to exist in an ensemble of structures, and because the 3D structure of these molecules is rarely known, there is not yet a consensus representational standard of per-residue accessibility for single-stranded nucleic acids. Ding et al. [17,18] implement probability of single-strandedness, when the weighted ensemble of likely structures is taken into account, as an accessibility criterion. However, use of their Sfold server, with batch jobs limited to 3500 bases, is not currently practical for a genome-scale survey of accessibility. Another approach to accessibility prediction is McCaskill's partition function approach [20] which can be used to compute base pair probabilities and summary pairing probability for any base. This approach is implemented in RNAFold [19], a component of the Vienna RNA package.

In this study, we chose to use the less physically rigorous approximation of probability of single strandedness as a simple fraction of predicted optimal and suboptimal structures in which a residue is found to be part of a single stranded structure, as computed by Mfold. Accessibility scores derived from MFold predictions have been used in limited studies of RNA structure focused on hammerhead ribozymes[30], antisense and siRNA targeting [22,31] and have been shown to be predictive in cases where some experimental measure of accessibility has been made[32]. While MFold-derived accessibility scores may not be completely optimal, they have been used with reasonable success to predict accessibility in the siRNA targeting context, and so we use MFold here.

### Shearing simulation

Random shearing of the target mixture is an approach that is often offered as a solution for the problem of target secondary structure. The actual content of a sheared mixture of DNA or RNA fragments is complex. Shearing breaks the molecule not in predictable locations, but in random locations that give rise to a distribution of fragments around an average fragment length. In order to simulate the effects of different degrees of shearing on structure formation and stability in a transcript, we picked fragments of 200, 100, or 50 bases in length, choosing the start position via a sliding window of 10 bases. Secondary structure prediction for all fragments derived from every transcript in the B. suis genome is computationally intensive and produces an extremely large amount of output. Since our initial goal was to determine how much the method would affect the number and type of secondary structures probes would be expected to bind the shearing simulation was performed for fragments derived from the 300 bp Ure-1A gene of *B. suis*. Secondary structure and thermodynamics were computed for each of these fragments individually.

## Authors' contributions

VGR participated in the design of the study, carried out the simulations and analysis, and drafted the manuscript. JWW participated in the design of the study and helped to draft the manuscript. CJG conceived of the study, participated in its design, coordinated the research and analysis, and drafted the manuscript.

**Table 1:** Stability of a sample transcript – full length target and sheared fragments Folding ΔG of target transcript and fragment molecules shown in Figure 8, at hybridization temperatures commonly used for long oligomer arrays.

**Molecule**	**^2^G, kcal/mole**			
	
	**42°C**		**52°C**	
	
	**DNA**	**RNA**	**DNA**	**RNA**
**70-mer Probe**	- 6.8	N/A	- 4.2	N/A
**Full Length Target**	- 85.9	- 188.4	-56.6	- 140.2
**200-mer sheared Target**	- 25.5	- 58.6	-15.9	- 41.6
**100-mer sheared Target**	-14.2	- 25.7	-9.6	-18.0
**50-mer sheared Target (not shown)**	- 6.1	-10.5	- 4.2	-7.3
